# Does the transparency of the counting system affect children's numerical abilities?

**DOI:** 10.3389/fpsyg.2015.00945

**Published:** 2015-07-07

**Authors:** Ann Dowker, Manon Roberts

**Affiliations:** ^1^Department of Experimental Psychology, University of OxfordOxford, UK; ^2^Worcester CollegeOxford, UK

**Keywords:** young children, number representation, estimation, cross-linguistic, counting system, Welsh bilinguals

## Abstract

The Welsh language uses a regular counting system, whereas English uses an irregular counting system, and schools within Wales teach either through the medium of Welsh or English. This provides the opportunity to compare linguistic effects on arithmetical skills in the absence of many other confounding factors that arise in international comparisons. This study investigated the hypothesis that language properties influence children's performance in certain numerical tasks by comparing the performance of 20 Welsh- and 20 English-medium Year Two pupils in non-verbal line estimations and transcoding. Groups did not differ on global arithmetic abilities, but the pupils taught through the medium of Welsh on average performed better in the non-verbal line estimation tasks than the English-medium group. This superiority was most apparent in comparisons involving numbers over 20: a result which was complicated by the fact that Welsh-medium pupils showed a lower range of error scores than the English-medium pupils. These results were thought to be related to the increased transparency of the Welsh counting system.

## Introduction

Comparisons of arithmetical performance of children in different countries have consistently shown significant differences (e.g., TIMSS, 1996; OECD, 2014). There are many possible cultural differences that may influence arithmetical development. These may include attitudes to mathematics or to academic skills in general; methods of mathematics education; the amount of time that is devoted to arithmetic teaching in school, and the economic or political situation of a country. The cultural issue that will be considered in the present article is language.

Children in Pacific Rim countries, such as China, Japan, and Korea, show superior performance in arithmetic (e.g., Fuson and Kwon, 1992; Miller et al., 1995; Miura and Okamoto, 2003), which is often attributed to the regularity of Asian counting systems. A regular counting system is purely multiplicative, meaning if the oral counting system corresponds closely to the written number word system, then children need only learn the digit names of one to nine and of multiples (i.e., 10, 100, 1000), to be able to say any number word up to 9999. Irregular counting systems on the other hand, such as English, include number words that do not show a one-to-one correspondence with the Arabic written system, such as teen numbers, e.g., “thirteen,” and multiples of ten, e.g., “twenty,” which consequently need to be learned separately. These differences in the degree to which the spoken and written number systems of languages coincide with each other have been suggested as an explanation for some of the results of international comparisons of arithmetical performance of children, as a regular counting system might facilitate children's learning to count to higher numbers, and might also increase their understanding of place value concepts.

Though systematic research on the subject began relatively recently, the idea that counting systems may influence arithmetical ability has been proposed for a long time. Two-hundred years ago, Edgeworth and Edgeworth (1798) posited the English language's highly irregular counting system as a possible disadvantage to English speakers when developing arithmetical skills. Although the cross-cultural differences that have been found in arithmetical performance could be due to differences in number word systems, the fact that some countries with transparent counting systems also show superior results in mathematics performance does not prove a causal relationship. Other cross-cultural differences, such as those mentioned above, could also be contributing to mathematical performance differences. In order to assess the impact of counting system on the development of numerical skills, it is important to rule out other differences as much as possible.

Studies of children living in Wales offer a promising solution to this problem. In Wales, 80% of children receive their school instruction in English-medium schools, while 20% receive their instruction in Welsh-medium schools. All of the children are living in the same area, in similar cultural environments, and all are studying mathematics in a similar fashion according to the National Curriculum of England, Wales and Northern Ireland. Thus, it is possible to study the specific effects of language on arithmetic, independently of other educational and cultural differences. The Welsh language in fact contains both an irregular counting system and a regular counting system, but only the regular counting system is used when teaching arithmetic in schools (Roberts, 2000). For example, whereas in English, “eleven” does not correspond to the written digits, in Welsh “un deg un” is used, which translates to “one ten one.” The Welsh term for “twenty-one” is “dau ddeg un” or “two tens one”, which represents the tens and units more transparently than the English term.

So far, few studies have compared numerical skills in Welsh children attending English- and Welsh-medium schools. One such study was conducted by Dowker et al. (2008), in which the performance of Welsh-speaking and English- speaking children living in Wales in a variety of number processing tasks was compared. The results showed that Welsh children demonstrated superior performance in comparing two-digit numbers, but the two groups did not differ in terms of arithmetic test performance. The authors concluded that such results indicate that linguistic effects affect specific aspects of arithmetic performance as opposed to more global effects (also see Mark and Dowker, 2015). This is consistent with the fact that arithmetic performance has often been shown to be comprised of numerous components, as opposed to being a unitary function (e.g., Dowker, 2005).

Most studies of cross-linguistic differences in numerical abilities, including Dowker et al.'s earlier (2008) study of Welsh children, have looked either at possible differences in arithmetical procedures, or on transcoding and related skills such as written number comparison, or both. If counting system effects are found on arithmetic or transcoding, this may either reflect effects on the ease of carrying out procedures in different counting systems, or differences in the internal representation of number by users of different counting systems. Relatively few studies have looked directly at whether children's internal representations of number are affected by the transparency of the counting system; and this is an important issue to resolve.

One way of studying the internal representation of number is to use the mental number line. Number line estimation tasks require participants to judge the position of a target number on a blank number line (e.g., Siegler and Booth, 2004; Booth and Siegler, 2006; Siegler et al., 2009). By calculating the discrepancy between the participant's estimate and the target number's actual location, number line estimation tasks can be used to assess participants' internal mental number line representation. Research has suggested that overall number sense revolves around a mental number line, and that children's mental number line representations correlate with and are casually linked to arithmetic performance (e.g., Siegler and Booth, 2004; Booth and Siegler, 2006, 2008; Link et al., 2014). Furthermore, a developmental shift has been consistently observed between the ages of 5 and 8 years, in which children rely increasingly on linear representations of numerical magnitudes as opposed to a reliance on logarithmic representations, with the shift being age dependent, and occurring for smaller scales before a linear representation of larger scales emerges (Booth and Siegler, 2008). Using non-verbal number line estimation tasks, in which the target number is presented visually rather than verbally, is particularly advantageous as makes it possible to assess linguistic effects on mental number line representations without the confounding factor of verbal comprehension, which might be separately influenced by language.

The few studies that have so far investigated the mental number line representations of children from different linguistic backgrounds have given somewhat conflicting results, though most have suggested at least some linguistic influence. Siegler and Mu (2008) found that Chinese kindergarten children performed better than their American counterparts on mental number line estimation tasks involving a number line spanning from 1 to 100. Laski and Yu (2014) found that both Chinese and Chinese-American children performed better than monolingual English-speaking American children, though they also found that children in China performed better on these tasks than Chinese-American children, suggesting that educational factors were more important than linguistic factors. By contrast, Muldoon et al. (2011) did not find such a difference between Chinese and Scottish 4-and 5-year-olds; and indeed when smaller number lines from 0 to 10 and 0 to 20 were included, the Scottish children performed better.

Helmreich et al. (2011) looked at another language group, with a counting system that is *less* transparent than most Europaean counting systems. German speakers have a counting system that has the potentially confusing property of inversion of tens and units: e.g., the number that is written as 24 is spoken as “vier und zwanzig” (twenty-four). Helmreich et al compared German- and Italian-speaking children's estimation accuracy in a non-verbal number line estimation task in which the number line spanned from “0” to “100.” The German children did indeed perform significantly worse than the Italian-speaking children on the mental number line task, though not in tests of global arithmetical ability.

The main aim of the current study was to extend the findings of Dowker et al. (2008) to investigate whether Welsh and English medium children in Wales would differ in the precision of their non-verbal number line estimation. In other words, does the language of school instruction affect the mental number line in children who are otherwise having similar cultural and educational experiences? Number line estimation tasks do not form part of the teaching syllabus in Wales, meaning children's performance in this task should be largely immune to teaching effects. Additionally, we wished to assess and compare the children's general arithmetical ability and their numerical transcoding abilities: their ability to read and write two-digit numbers.

The performance of 20 Welsh-medium and 20 English-medium children was compared regarding the British Abilities Scale (BAS) Number Skills test, writing to dictation, reading numbers aloud, and a non-verbal line estimation task, which included number lines from 0–20 and 0–100.

We predicted that the Welsh-medium children would score higher on the number line estimation tasks, and that this would be especially true of the number line from 1 to 100, as this places greater demands on the ability to represent multi-digit numbers, for which a transparent counting system would provide an advantage. On the basis of Dowker et al.'s (2008) findings about Welsh-medium children's better performance on two-digit number comparison, we also predicted that the Welsh-medium children would perform better on the tasks involving reading and writing numbers. However, we predicted that there would be no difference between the two groups in BAS Number Skills test performance, based on Dowker et al.'s (2008) earlier findings and their suggestion that linguistic effects on mathematics may be specific rather than global.

## Method

### Ethical approval

Ethical approval for this study was obtained from Oxford University's Central University Research Ethics Committee.

### Participants

Forty children, drawn from the Year Two classes of two state primary schools in Cardiff and one state primary school from the Rhondda Cynon Taf area, took part in the study. The data from all participants were included in the analysis. Written consent was obtained from all parents or guardians. One Cardiff-based school taught through the medium of Welsh, from which 20 children (10 girls) took part. The other two schools were English-medium schools, and 10 participants from each school (20 in all, including 14 girls) took part. All the children in the Welsh- medium schools were taught exclusively through the medium of Welsh, but 13 of the 20 children spoke English as a first language. Though taught through different languages, mathematics teaching followed exactly the same curriculum in the three schools. The 20 Welsh-medium children were compared with the 20 English- medium children. All were tested at the same time of their school year, but they turned out to be somewhat different in age. The mean age of the Welsh-medium school pupils was 6 years and 5 months (*SD* = 0.30; range 73–85), and the English-medium school pupils 6 years and 7 months (*SD* = 0.35; range 73–84). The age difference between the two groups was significant [*t*_(38)_ = 2.38, *p* = 0.022, *d* = 0.75]. All children had normal or normal-to-corrected vision.

### Tasks and procedure

The children completed four tasks: the BAS Number Skills test, which is a standardized test that assesses written calculation (Elliott et al., 1997), two transcoding tasks (writing to dictation and reading aloud), and a non-verbal number line estimation task.

In the *writing Arabic numbers to dictation task*, participants were required to write down 32 different Arabic numbers (two single-digit, 10 double-digit, and 20 3-digit numbers) that were presented verbally one by one by the experimenter.

In the *reading Arabic numbers aloud task*, participants were required to read aloud 32 different Arabic numerals (two single-digit, 10 double-digit, and 20 three-digit numbers) that were presented on a computer screen one by one. In both transcoding tasks, items were scored with a 0 for every incorrect answer, and 1 for every correct answer.

The *number line task* was a pen and paper task that required participants to estimate the position of a visually presented number (as opposed to verbally presented) on an empty number line, without counting or using any other strategy other than estimation. The number lines were 10 cm long, and labeled with “0” on the left end, and “20” or “100” on the right end. Each number to be estimated was presented centrally above each empty individual number line in Arabic notation. Participants estimated the position of the numbers 12, 1, 13, 4, 15, 19, 7, 17, and 5 on 0–20 number lines, and the position of the numbers 27, 2, 64, 35, 7, 13, 99, 75, 47, 3, 11, 82, 95, 9, 17, 6, 18, and 53 on 0–100 number lines. Before beginning the task, participants were presented with an orienting problem for each of the two different number lines, where they were required to estimate the position of 10 on the 0–20 number line, and 50 on the 0–100 number line for practice purposes.

The children were tested in one-to-one single sessions with the experimenter. The tasks were explained and conducted in Welsh for the Welsh-medium education children and in English for the English-medium education children. Each trial was presented sequentially, and no feedback on performance was provided for any of the trials, including the practice trials.

The data were analyzed using IBM SPSS 20.

## Results

### Overall mean scores

The mean raw score on the British Abilities Scales Basic Number Skills test (henceforward referred to as BAS) was 8.25 (s.d. 3.24) and the mean standard score was 107.98 (s.d. 11.9). The mean score for the Reading Aloud test was 20.98 (s.d. 8.42) and the Writing test was 19.75 (s.d. 7.95).

To obtain number line estimation scores, the distance between the true position of the number that was presented and the position of the number corresponding to the child's estimate on the number line was measured to the nearest millimeter. These deviation measures were then averaged for each participant individually to give two mean estimation error scores; one score for the 0–20 number line estimations, and one for the 0–100 number line estimations. These were also averaged to obtain an overall mean estimation error for each participant.

The mean estimation error score for the 0–20 number line was 12.12 mm. (s.d. 9.43) and the mean estimation error for the 0–100 number line was 20.36 mm. (s.d. 7.15). The overall mean estimation error score was 17.58 (s.d. 6.98).

### Correlations between age, BAS scores and other measures

A correlation table is given in the Supplementary Material.

Pearson product-moment correlations were carried out between Age in months and the other measures. Age did not correlate significantly with the BAS raw score or standard score, nor with the estimation error measures, though there was a trend toward a significant negative correlation with errors for the 0–100 number line, i.e., for older children to perform slightly better [*r*_(38)_ = −0.295; *p* = 0.065]; but it did correlate very significantly with Reading Aloud [*r*_(38)_ = 0.53; *p* < 0.001] and Writing [*r*_(38)_ = 0.46; *p* = 0.003].

Pearson product-moment correlations were carried out between BAS raw score and the other measures. The BAS raw score showed a significant negative correlation with estimation errors overall [*r*_(38)_ = −0.439; *p* = 0.005] and with errors for the 0–100 number line [*r*_(38)_ = −0.479; *p* = 0.002], though it did not correlate significantly with errors for the 0–20 number line [*r*_(38)_ = −0.25; *p* = 0.12]. It also correlated significantly with Reading Aloud [*r*_(38)_ = 0.69; *p* < 0.001] and Writing [*r*_(38)_ = 0.73; *p* < 0.001]. Correlations between the BAS standard score and the other measures were very similar to those between the BAS raw score and the other measures.

The overall estimation error score showed a significant negative correlation with Reading Aloud [*r*_(38)_ = −0.6; *p* < 0.001] and Writing [*r*_(38)_ = 0.49; *p* < 0.001].

For a more detailed list of correlations, see the Supplementary Material.

### Comparison between groups: analyses of covariance

As the language groups differed significantly in Age, and as Age correlated significantly with some measures, Analyses of Covariance were carried out with Age as a covariate.

To compare the Reading Aloud and Writing scores of the Welsh- and English-medium groups, two univariate ANCOVAs were conducted with language as the fixed factor, age as a covariate, and the Reading and Writing scores as dependent variables. Group differences did not approach significance for either task.

To compare the estimation errors of the Welsh- and English-medium groups, three univariate ANCOVAs were conducted with language as the fixed factor, age as a covariate, and the three different mean estimation error scores as dependent variables. The ANCOVA revealed no significant group difference for the 0–20 number line estimation errors [*F*_(1, 37)_ = 2.77; *p* = 0.11; partial eta^2^ = 0.05], but did reveal a significant difference for the overall estimation error score [*F*_(1, 37)_ = 4.36, *p* = 0.044; partial eta^2^ = 0.11], and a borderline significant difference for estimation errors in the 0–100 number line task, [*F*_(1, 37)_ = 3.77, *p* = 0.06; partial eta^2^ = 0.092]. Estimation errors were lower in the Welsh-medium group for all these tasks, though the difference only approached significance for the 0–100 task (*M* = 19.05, *SD* = 5.52 for the Welsh-medium pupils compared to *M* = 21.69, *SD* = 8.42 for the English-medium pupils); and reached it for the overall estimation error score (*M* = 16.06, *SD* = 4.66 for the Welsh-medium pupils and *M* = 19.1, *SD* = 8.57 for the English-medium pupils).

### Further analyses of estimation error scores

As the 1–100 line included both comparisons of numbers over 20 and numbers under 20, analyses were carried out on mean errors for both types of number separately, to elucidate whether group differences related to the entire number line, or just to the larger numbers. For numbers under 20, there was no significant group difference at all [*F*_(1, 37)_ = 0.844, *p* = 0.89; partial eta^2^ = 0.001]. However, for numbers over 20, the group difference was significant [*F*_(1, 37)_ = 9.14, *p* = 0.003; partial eta^2^ = 0.274]. The Welsh-medium pupils had a mean estimation error score of 14.67 (*SD* = 4.88) as compared with 21.09 (*SD* = 13.5) for the English-medium pupils.

Since the standard deviation was higher in the English than the Welsh group for numbers over 20 on the 1–100 line, analysis of variance may not be a fully adequate measure; and non-parametric analyses were also carried out. A Kolmogorov-Smirnov independent-samples test failed to reach significance (*p* = 0.172); while a Moses Test of Extreme Reaction showed a significant difference between the ranges of the two groups, with a higher range in the English group (*p* = 0.02).

For the 00–20 number line, analyses were carried out on mean errors for teen numbers vs. numbers below 10. To compare the estimation errors of the Welsh- and English-medium groups, two univariate ANCOVAs were conducted with language as the fixed factor, age as a covariate, and the two different mean estimation error scores (for numbers under 10 and over 10 on the 1–20 number line) as dependent variables. The ANCOVA revealed no group difference even approaching significance for numbers under 10 [*F*_(1, 37)_ = 0.005; *p* = 0.95], but did reveal a significant difference for numbers over 10 [*F*_(1, 37)_ = 5.43, *p* = 0.025; partial eta^2^ = 0.13]. For the numbers over 10, Welsh-medium children made lower estimation errors (*M* = 11.64; *SD* = 10.39 for the Welsh-medium group and *M* = 18.76; *SD* = 13.62 for the English-medium group.

## Discussion

This study aimed to extend Dowker et al.'s (2008) study of Welsh children, and to examine the role of language in children's transcoding skills and non-verbal number line estimations by comparing two groups of school pupils for which cultural and educational variables were not strong confounding variables.

The results suggest that the transparency of the counting system may indeed have an influence on children's number representation, far more than on arithmetical skills. There was no evidence in this study for better arithmetic or transcoding in the Welsh-medium children. The two groups performed similarly in the BAS standardized test, and in the reading and writing of numbers.

However, after controlling for age, the Welsh-medium children performed better on the number line estimation task. This is congruent with the findings of Siegler and Mu (2008), Laski and Yu (2014), and Helmreich et al. (2011), indicating that the transparency of the counting system may influence not only the ease of carrying out procedures with numbers (although in the present study there was no evidence for this), but their representations of numbers.

This is particularly striking, because in this study there were no apparent environmental differences between the groups, other than the linguistic ones. The children attended apparently similar schools, and were all studying mathematics according to the same curriculum. It should be noted that the majority of the Welsh-medium children spoke English at home, which makes any effect of school instruction medium even more striking. While it is not possible completely to rule out the influence of some non-linguistic characteristics of a particular school or teacher, this seems unlikely, especially as the groups did not differ on the standardized arithmetic test.

The group differences varied both with the nature and extent of the number line and with the size of the numbers involved in particular tasks. The Welsh-medium group made lower estimation errors, and this difference was significant overall and approached significance for the 0–100 number line, but not for the 0–20 number line. This is consistent with the prediction that language would be likely to affect number line estimation more for multi-digit numbers. This prediction was indeed supported by further analyses of estimates for different sizes of numbers within the 0–20 and 0–100 number lines. The groups differed on the 0–20 number line for their estimates of teen numbers, but not of numbers under 10. On the 0–100 number line, they differed significantly for numbers over 20, but not at all for numbers from 0 to 20 (single digit or teen). Thus, whether the groups differed for teen numbers or not seemed to differ according to the surrounding numerical context, but there is more evidence that the groups differed for numbers over 20, and did not differ for numbers under 10. In other words, the precision of numerical representations was most affected by language for numbers that required an understanding of the relationship between tens and units, which appears to be facilitated by a transparent counting system. These representations were also those most related to arithmetical performance: the BAS Arithmetic test correlated significantly with the error score on the 1–100 number line but not the 0–20 number line.

There is, however, an important qualification with regard to the statement that the groups differed significantly for numbers over 20. The *range* of error scores was significantly higher for the English medium pupils. Presumably as a result of this, a non-parametric comparison between the language groups for this group of numbers failed to reach significance. The greater range of scores for the English medium group is intriguing in itself. One possible explanation may be that the transparent counting system of Welsh constrains the strategies for estimation, while the less transparent English system provides fewer constraints and cues, leading to greater variability in strategies and thereby in scores. A yet more interesting possible explanation is that the transparent counting system constrains representation as such, and that this is far more variable in a more opaque counting system. Clearly, more research needs to be done, involving a wider variety of representational tasks and a larger set of transparent and opaque counting systems.

The hypothesis that reading and writing two-digit numbers would be affected by the language group was not supported at all. It may be that these skills, which are school-taught, depend more on specific teaching, which would be similar in the Welsh- and English-medium schools, and do not depend strongly on internal numerical representations. Although this finding might seem to conflict with the earlier results of Dowker et al. (2008) that indicated that Welsh-medium children were better than English-medium children at dealing with two-digit numbers, the tasks were rather different. The task in Dowker et al.'s (2008) study involved reading *and comparing* two-digit numbers; and it may be that the comparison element of the task was more dependent on internal representations.

Intriguingly, what did correlate quite strongly with the number reading and writing tasks was chronological age, even though the age range in this study was restricted and the children were all tested at the same time in their school year. Further research needs to be done to see whether maturation or perhaps some specific aspect of home experience is particularly important in the development of these skills. The finding that young children can show a significant age correlation, even within a quite restricted age range, with some numerical abilities but not others is congruent with earlier studies with somewhat younger children (Dowker, 2008), and clearly needs further exploration.

Thus, the results imply that greater transparency in a language's counting system may lead to the developmental shift in children's mental number line representation (that is, the shift from a logarithmic representation to a linear representation) occurring at an earlier age.

Given that previous research has documented this representation's link with overall arithmetic ability, the advantage of being taught arithmetic through the medium of a language that utilizes a regular counting system may be more widespread than previously thought. It would be interesting to carry out longitudinal studies to investigate whether this advantage persists longitudinally, and whether it predicts any other aspects of later arithmetic. The Welsh-medium children did better at the number line estimation tasks; these tasks correlated with a standardized arithmetic test; but the Welsh- medium children did not at this stage do better at the standardized arithmetic test. Might the number line advantage correlate with standardized arithmetic test differences, or with differences in more specific aspects or arithmetic later on? Cross-sectional studies of a wider variety of age-groups would also yield interesting information about how language effects on numerical skills might change with age.

Though the results do suggest that the transparency of a language's counting system has an effect on some aspects of number processing, some caution is needed to drawing extremely strong conclusions, as the groups, though from similar backgrounds, were not matched in advance on all possible factors, and in particular there was an unexpected small but significant difference in age. This was controlled for in the analyses by including age as a covariate, as age did show a surprising level of correlation with some measures; but this is not a completely satisfactory solution, and future studies should ensure matching for age.

Moreover, direct measures of proficiency in the two languages were not obtained. Although the English-medium children studied Welsh formally as a second language at school, they may not have been bilingual in the same sense as children who speak English at home and Welsh at school. At present, we cannot be certain about the extent to which group differences are the result of general proficiency in Welsh; Welsh medium mathematics instruction in particular; or the experience of bilingualism. It would be desirable for future studies to explore the issue further. In particular, it would also be interesting to study Welsh-English balanced bilinguals, and to investigate whether different results would be obtained when testing the same children in Welsh vs. English.

These results may have some implications for educational practice. As stated in the Introduction, there is evidence that the development of linear representations of numerical magnitude contributes to arithmetical development (Booth and Siegler, 2008). The present study also supports a few earlier studies in suggesting that this development may be facilitated by regular counting systems and impeded by irregular counting systems. Perhaps English-medium schools, or any schools that teach through the medium of a language that has an irregular counting system, could investigate ways of helping children generate linear representations of number. For example, one way of achieving this might be is through the playing of board games in which the counters are moved linearly across equidistant spaces, thus providing a transparent representation of numerical magnitude (Siegler and Ramani, 2009).

The main conclusion of this study is that the regularity and transparency of the Welsh counting system may help children not only in learning the correspondence between written and oral representations of number but also in the development of non-verbal numerical magnitude representations. Therefore, the influence of language should be considered when teaching number processing, especially when teaching children who struggle with mathematics.

### Conflict of interest statement

The authors declare that the research was conducted in the absence of any commercial or financial relationships that could be construed as a potential conflict of interest.
